# Understanding the health-related quality of life and treatment-related side-effects in patients who have been in remission from testicular cancer for 12–24 months

**DOI:** 10.3389/fruro.2023.1174626

**Published:** 2023-07-31

**Authors:** Walter Cazzaniga, Janette Kinsella, Adam Kieran Pearce, Masood Moghul, Louis Fox, Mieke Van Hemelrijck, Alison Reid, Robert Huddart, David Nicol

**Affiliations:** ^1^ Department of Uro-Oncology, The Royal Marsden NHS Foundation Trust, London, United Kingdom; ^2^ Urology Department, Royal Brisbane and Women’s Hospital, Brisbane, QLD, Australia; ^3^ Centre for Cancer, Society, and Public Health, King’s College London, London, United Kingdom; ^4^ Institute of Cancer Research, London, United Kingdom

**Keywords:** testicular cancer, PROMs, side effects, health-related quality of life (HRQoL), testicular cancer treatment

## Abstract

**Introduction:**

Despite the excellent long-term prognosis after treatment for testicular cancer (TCa), therapy-related side effects can be persistent and severe. The aim of this study was to determine the nature and prevalence of post-treatment symptoms and their impact on health-related quality of life (HRQoL) in TCa patients 12 to 24 months after treatment.

**Materials and methods:**

Cross-sectional, single-center study. All patients who were aged 18 and over, had completed TCa treatment 12–24 months previously and had no evidence of disease recurrence were considered eligible. Participants were stratified into four groups: 1) orchidectomy only; 2) orchidectomy and single dose adjuvant carboplatin; 3) multi-agent induction chemotherapy (CBOP-BEP, BEPx3 or x4, or Epx4 regimens); and 4) post-chemo retroperitoneal lymph node dissection (PC-RPLND). Eligible patients were asked to complete the EQ-5D-5L questionnaire and the EORTC QLQ-TC26 questionnaire. We performed a thematic analysis of free-text commentary to evaluate the sensitivity of PROMs used across the treatment groups. Descriptive results were reported. For categorical variables, numbers and percentages were used, and for continuous variables median and IQR values were used.

**Results:**

The EQ-5D-5L questionnaire showed that patients treated with orchidectomy only and orchidectomy and adjuvant carboplatin experienced only minor physical medium- to long-term side-effects. In contrast, more intensive treatment regimens, such as multi-agent chemotherapy or PC-RPLND, were associated with a higher burden of medium- to long-term side-effects. Similar results were obtained with the EORTC QLQ-TC26 questionnaire.

**Conclusions:**

This study reports the medium- to long-term HRQoL and side effects of TCa treatments, using both EQ-5D-5L and EORTC QLQ-TC26 questionnaires, and identifies possibly “unasked” questions from a patient perspective in relation to supportive care needs following TCa treatment. This information will help clinicians to better understand the consequences of treatment and in turn provide better patient counseling before treatment.

## Introduction

Testicular cancer (TCa) is the most common solid organ malignancy affecting young men, and represents 5% of all urological cancers (1, 2). Its treatment typically involves a plethora of treatments (3) depending on stage and histologic sub-type.

The specific consideration of patient experiences may be less of a priority due to an incomplete understanding of what these experiences precisely are. Patients may have difficulty articulating their experiences in treatment discussions and feel an obligation to concur with clinician advice when more than one option exists (4–7).

In this study we observe and compare the prevalence of post-treatment symptoms and health-related quality of life (HRQoL) 12–24 months following treatment in patients treated in a UK tertiary TCa center. We also performed a thematic analysis of free-text commentary to evaluate the sensitivity of patient-reported outcome measures (PROMs) used across the treatment groups.

## Materials and methods

### Study design

The study had a mixed-methods cross-sectional design, incorporating the collection of quantitative data from PROMs and qualitative data from free-text responses (the two questions asked of patients).

### Participants

The sample comprised 73 TCa patients, who were identified and recruited through a review of weekly TCa multi-disciplinary team meetings (MDTs) and Royal Marsden testicular cancer clinics. All patients (aged 18 and over) who had completed one of the following treatments for TCa and had no evidence of disease recurrence were considered eligible: 1) orchidectomy only, henceforth the orchidectomy only group; 2) orchidectomy and single-dose adjuvant carboplatin, henceforth the orchidectomy and carboplatin group; 3) multi-agent induction chemotherapy (CBOP-BEP, BEPx3 or x4, or EPx4 regimens), henceforth the multi-agent chemotherapy group; and 4) post-chemo retroperitoneal lymph node dissection (PC-RPLND), henceforth the PC-RPLND group. Patients were excluded from the study if they had undergone high-dose chemotherapy with stem cell transplant, were unable to read and write in English, or were regarded as lacking the capacity to provide informed consent. To evaluate the medium- to long-term side effects of treatment, all patients included in the study had completed TCa treatment 12 to 24 months before the study.

### Questionnaire design and content

A questionnaire was developed to include two PROMs designed to measure HRQoL: the EQ-5D-5L and EORTC QLQ-TC26. The EQ-5D-5L is a widely used generic instrument for assessing HRQoL (8, 9). It comprises two parts, with the first part being a “descriptive system” in which respondents are asked to grade five descriptors (mobility, self-care, usual activities, pain/discomfort, and anxiety/depression) based on the severity of problems. The second part is a visual analog scale, in which respondents are asked to rate their health on that day on a scale from 0 (worst health imaginable) to 100 (best health imaginable) (10). The EORTC QLQ-TC26 questionnaire is a validated measure which has previously been used to study post-treatment side effect severity and prevalence in TCa patients, particularly in the chemotherapy setting. The QLQ-TC26 employs a symptom severity score (0 = not at all, 1 = a little, 2 = quite a bit, and 3 = very much) and comprises seven multi-item scales (treatment side effects, treatment satisfaction, future perspective, communication, sexual activity, functioning, and enjoyment) and six single items (job and education problems, physical limitations, family problems, infertility, body image problems, and testicular transplant satisfaction).

Patients responding to the survey were also invited to describe any symptoms/problems that are not addressed/recognized as sequelae of treatment in the PROMs questionnaires. They were also asked to describe the aspect of TCa that had had the greatest impact on their lives, with the aim of providing an in-depth understanding of the supportive care needs of patients that was framed by their own perspectives and priorities. Those who opted to complete the free-text component were provided with two questions: (1) “Are there any symptoms or problems caused by testicular cancer treatment that affect you now that have not been mentioned or well described in this form?”, and (2) “Which single aspect of testicular cancer treatment has had the greatest impact on your quality of life and why?” (11).

### Data analysis

Inductive thematic analysis of the questionnaire free-text answers was undertaken whereby all datasets were read and re-read by a single researcher (NK) so that emerging themes (“funneling”) (12) could be identified. These key themes were extracted and described using a narrative analysis technique (31). These themes were then reviewed by a second researcher (WC). Statistical tests were conducted using R statistical software (version 3.4.3).

### Ethics and regulatory governance

Regulatory and ethics committee approval was granted by The Royal Marsden NHS Trust’s Research and Development Committee (Ref. No CCR4937). The study was conducted in accordance with the Declaration of Helsinki (as revised in 2013).

## Results

### Quantitative data analysis

#### EQ-5D-5L

**Figure 1:**
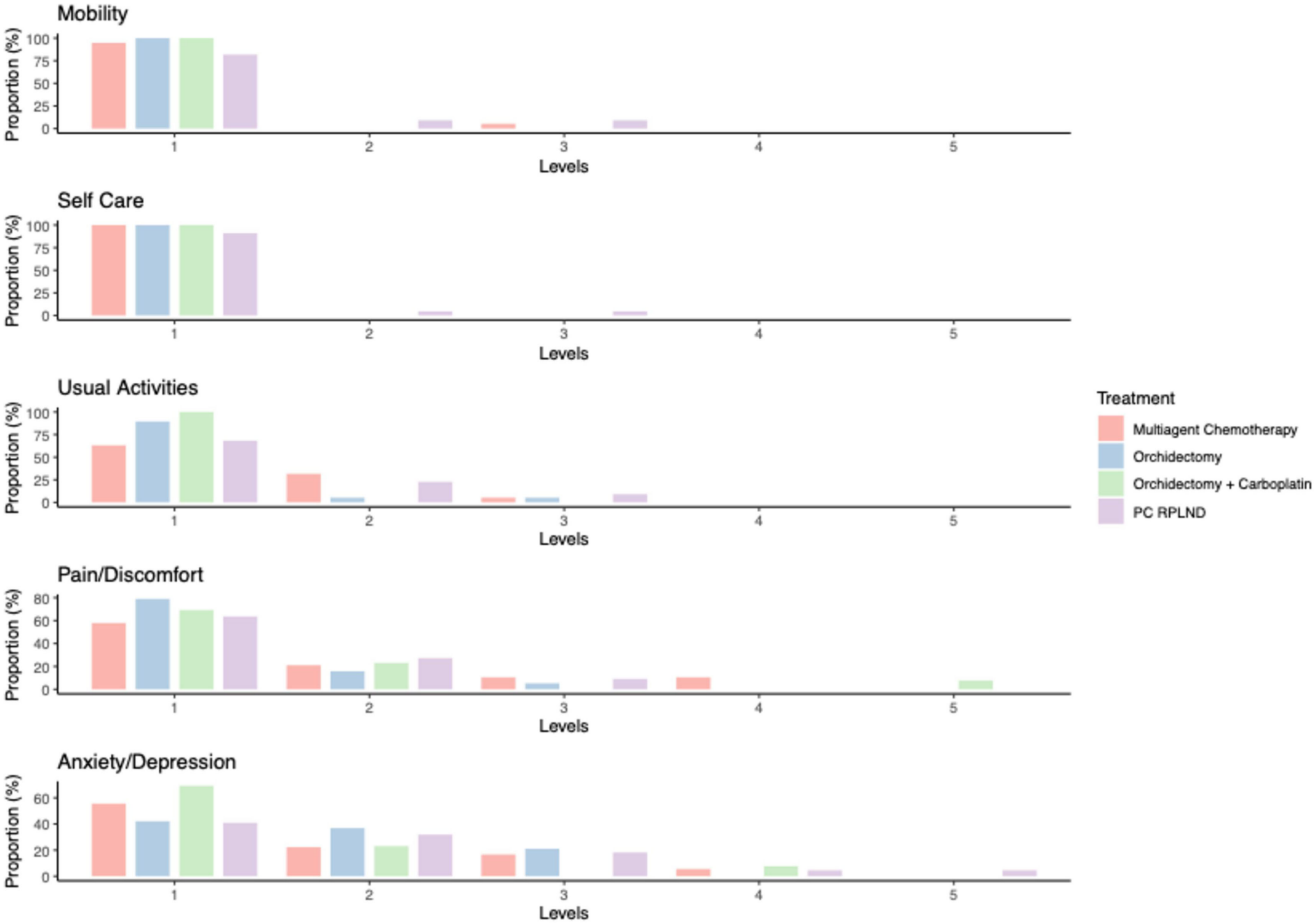
Bar plot reporting the results from the ED-5D-5L questionnaire. Levels: 1 = no problem, 2 = slight problem, 3 = moderate problem, 4 = severe problem, and 5 = extreme problem.

Analysis of the “descriptive system” portion of the EQ-5D-5L questionnaire showed that patients in the orchidectomy only and orchidectomy and carboplatin groups experienced few physical medium- to long-term side effects. Only a small proportion of patients within the orchidectomy only group reported a “slight” (1 out of 19 patients; 5.3%) or “moderate” (1 out of 19 patients; 5.3%) degree of impairment when carrying out their usual activities. Four patients (21.1%) in the orchidectomy only group reported a “slight” to “moderate” degree of pain/discomfort after treatment. This was also observed within the orchidectomy and carboplatin group (3 out of 13 patients; 23.1%), with one patient (7.7%) reporting severe pain/discomfort (**Supplementary Table 1**; **Figure 1**).

In contrast, more intensive treatment regimens, such as multi-agent chemotherapy or PC-RPLND, were associated with a greater burden of medium- to long-term side effects. In particular, 4 out of 22 PC-RPLND patients (18.2%) reported “slight” to “moderate” mobility impairment (Q8), 2 out of 22 patients (9%) reported problems in the self-care domain (Q9), and 7 out of 22 patients (31.8%) reported problems when carrying out their usual activities (Q10). Multi-agent chemotherapy alone had less impact on the mobility (1 out of 19 patients; 5.3%) and self-care domains (0 out of 19 patients; 0%), and seven out of 19 patients (36.9%) in this group reported a “slight” to “moderate” degree of impairment when carrying out their usual activities. Both treatments were associated with significant rates of medium- to long-term pain/discomfort; these symptoms were reported by 8 out of 19 patients (42.1%) in the multi-agent chemotherapy group, and 8 out of 22 patients (36.4%) in the PC-RPLND group (**Supplementary Table 1**; **Figure 1**).

With respect to the emotional burden of treatment, anxiety domain results were comparable across all treatment groups, with the exception of the orchidectomy and carboplatin group. Over half of patients treated with orchiectomy only (13 out of 19 patients; 57.9%) reported anxiety/depression; this was similar to results for the multi-agent chemotherapy group (8 out of 19 patients; 44.5%) and the PC-RPLND group (13 out of 22 patients; 59%). In the orchidectomy and carboplatin group, the rate of reported anxiety was much lower (4 out of 13 patients; 30.8%), with the majority reporting being only “slightly” anxious (**Supplementary Table 1**; **Figure 1**).

**Figure 2:**
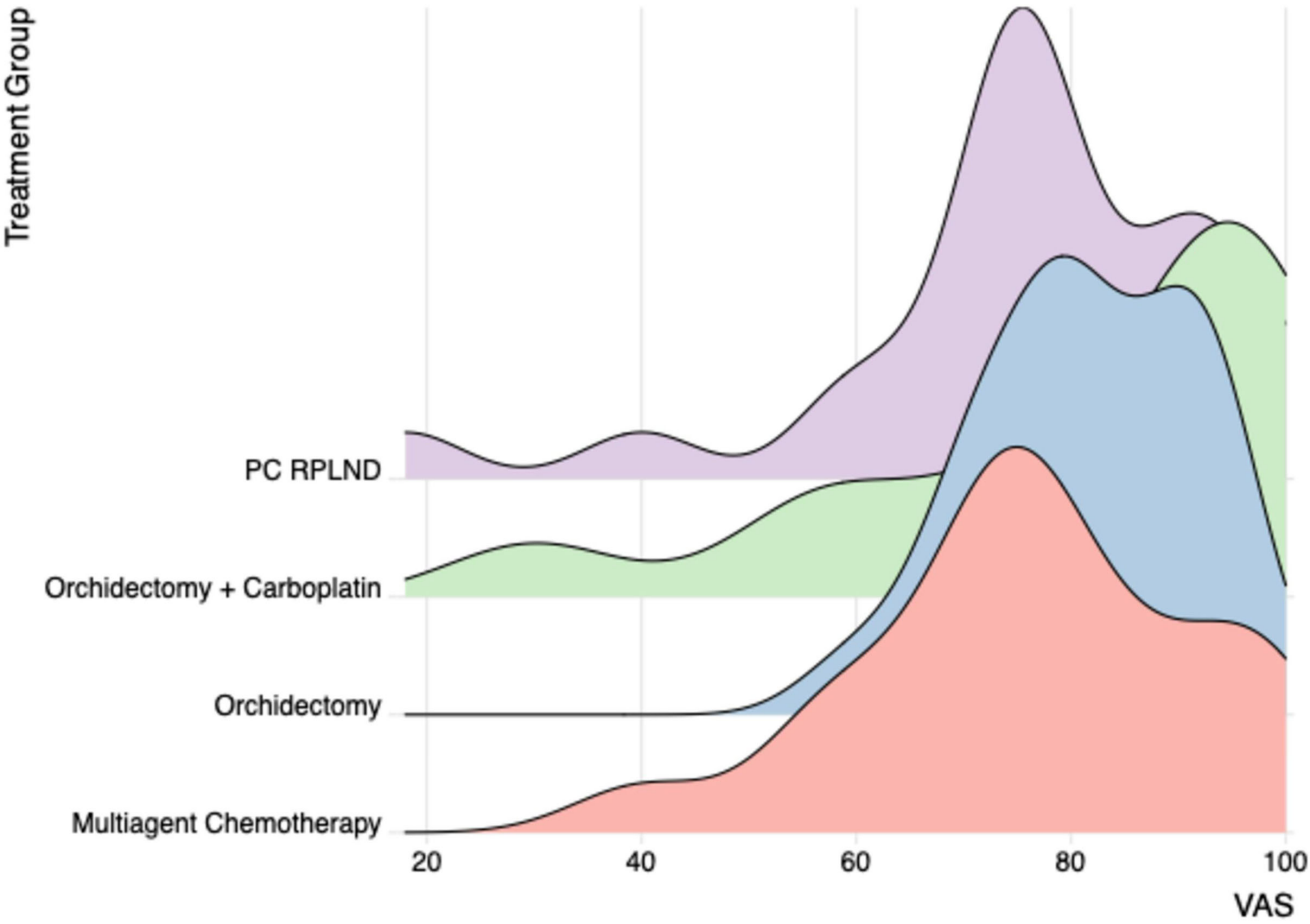
VAS obtained by ED-5D-5L questionnaire for the different treatment groups. VAS, visual analog scale.

Findings from analysis of the VAS data were consistent those found from the “descriptive system”, in the sense that men in the orchidectomy only and orchidectomy and carboplatin groups reported a higher self-perceived health status score than those undergoing more intensive treatment regimes (**Supplementary Table 2**; **Figure 2**).

#### EORTC QLQ-TC26

With EORTC-QLQ TC26, both the multi-agent chemotherapy and PC-RPLND groups had a similar side-effect profile. Specifically, both treatments induced a degree of perceived muscle loss and reduced strength, with these being reported by 13 out of 19 patients (68.4%) and 17 out of 22 patients (77.3%) in the multi-agent chemotherapy and PC-RPLND groups, respectively. The most commonly reported side effects in the multi-agent chemotherapy group were “tingling or numbness in extremities” (13 out of 19 patients; 68.4%), “Raynaud’s phenomenon” (10 out of 19 patients; 52.6%)”, “tinnitus” (7 out of 19 patients; 38.9%), “shortness of breath” (9 out of 19 patients; 47.4%), and “problems with taste and smell” (6 out of 19 patients; 31.6%). Over half of the surgically managed patients in the PC-RPLND group reported “problems with ejaculation” (14/22; 63.6%) and “concerns with body image” (12/22; 54,6%) (**Supplementary Table 3**).

The orchidectomy only and the orchidectomy and carboplatin groups shared a similar side-effects profile with the exception of questions Q17.1 and Q17.2, which addressed the psychological impact of the treatment. In the orchidectomy and carboplatin group, 8 out of 13 (61.5%) patients reported not feeling uncertain about the future, compared with 6 out of 19 patients (42.9%) in the orchidectomy only group (**Supplementary Table 3**).

### Qualitative data analysis of additional free-text questions

The free-text responses to question 1 (“Are there any symptoms or problems caused by testicular cancer treatment that affect you now that have not been mentioned or well described in this form?”) revealed a range of recurring recollections, feelings, and opinions, described below according to the following themes: (1) shortness of breath (SOB), (2) pain, (3) body image, (4) cognitive impairment, and (5) anxiety. **Supplementary Table 4** summarizes the key findings from the responses.

#### Shortness of breath

One-third of patients who offered free-text commentary felt that although the EORTC QLQ-TC26 asked about severity of SOB [(1) not at all, (2) a little, (3) quite a bit, and (4) very much], this did not adequately reflect either the breadth or context of the problem. Patients described their SOB in a variety of ways: “feeling breathless while resting”, having “weak lungs”, “finding it so hard to breathe”, or being “chesty in the mornings”.

#### Pain

The most common theme described by participants as being poorly defined by the PROMs questions was “pain”. Descriptions of pain ranged from “discomfort in wound site from orchiectomy” to a “slight pain/discomfort when ejaculating” to “pain daily at night”, suggesting that the patients who had commented (i.e., 50% of respondents) were unhappy with the binary format of the severity scoring system associated with the EORTC QLQ-TC26.

#### Body image

Body image was mentioned by one-quarter of respondents. The comments on this issue included “I am a bit self-conscious about the prosthesis”, “I was left with permanent pigmentation on my body after chemotherapy”, “my toe and finger nails are damaged”, and “I suffered from incomplete regrowth of hair”. Once again, this suggests that body image is an issue that is too complex to be adequately articulated using a binary assessment score.

#### Cognitive impairment

Cognitive impairment was reported by respondents who had received chemotherapy (i.e., those in the multi-agent chemotherapy, RPLND, and orchidectomy and carboplatin groups). Participant responses included “It was 1 year after chemo before I felt ‘normal’ and back to my old self”, “I suffered from mild cognitive impairment for almost a year”, “Memory recall was challenging for example I could not remember people’s names”, and “My concentration and speed of thought is not good”.

#### Anxiety

This theme, which was the most common across respondents (reported by 80% of respondents), had two discrete subthemes: (a) anxiety associated with cancer recurrence and (b) heightened anxiety associated with formal follow-up.

*a. Anxiety associated with cancer recurrence*

Many men described the daily psychological impact associated with the fear of cancer recurrence as being debilitating. One commented: “I have a 2-year-old and another baby due in January 2019 (conceived 18 months after treatment—yey)! and I worry about long term life expectancy”. Another reported having “the lingering thought that it may return”, with another suggesting that “not knowing the true cause and the likelihood of it returning is stressful”.

*b. Heightened anxiety associated with formal follow-up*

Anxiety around the time of cancer follow-up appointments was also described by many as preventing them from moving on. Comments included “when I go to the cancer center for check-ups I see people suffering infinitely worse than I did. So I almost feel like I do not have the right to talk about it or to acknowledge it happened to me, but it did!”, and “the fact you had it once always makes you anxious but especially during check-up time. So it’s more of a mental impact than physical”.

Responses to the second free-text question (“Which single aspect of testicular cancer treatment has had the greatest impact on your quality of life and why?”) are described across the following themes: body image and fatigue (**Supplementary Table 5**).

#### Emotional impact

Participants described the emotional impact of cancer as presenting itself in a myriad of ways. One man suggested “I suffer from claustrophobia now”, and another commented that “whilst I’m not a worrier and have a healthy attitude to getting on with it in life, I do wonder how if at all, how the chemotherapy has affected me”. Another suggested that their ongoing anxiety arose from the fear of cancer recurrence: “I had my left testicle removed and around the groin of my right one, it feels knotty I think this might be my lymph glands. It has been checked out but still causes me anxiety”.

#### Body image

Across all treatment groups, men described body image issues as having a significant impact on a daily basis. One patient said that “since the chemo I cannot be clean shaven or have short hair, as I cannot look at myself in a mirror, it reminds me of being ill”.

Others suggested that treatment had changed their body habitus: “I seem to have developed fatty chest tissue which I am very self-conscious about”, and “I am self-conscious about the look of the prosthesis”.

#### Fatigue

Men in the PC-RPLND and orchidectomy and carboplatin groups described “a lack of energy and poor general fitness that lingered”. They described it as “hard to get back into exercise”, with one saying “only now almost 2 years after diagnosis have I managed to start doing proper regular exercise”. In the other groups, patients commented on “a lack of energy resulting in inactivity and weight gain”.

## Discussion

In this study, analysis of the highly structured PROMs measures (ED-5D-5L and EORTC QLQ-TC26) combined with the free-text commentary provided insights into possibly “unasked” or poorly defined quality-of-life issues (according to patients) and into the single aspect of TCa that has the greatest impact on their lives.

The multimodal nature of TCa treatment exposes patients to a plethora of possible side effects that might significantly impact their quality of life at a very young age. Studies reporting on the most frequently occurring side effects identify them by treatment modality; multi-agent chemotherapy treatments most commonly cause hearing loss, tinnitus, peripheral neuropathy, Raynaud-like phenomena, and infertility (13), with the status at 1 year post-treatment likely to reflect long-term morbidity (4). Impaired ejaculation frequently occurs following RPLND (14), and radiation treatment may cause long-term intestinal problems, radiation-induced secondary malignant neoplasms, and muscle twitching (15, 16). In addition, fertility concerns, body image issues, and sexual dysfunction (including impaired ejaculation and decreased sexual satisfaction) are intrinsic themes of life after TCa treatment as the patient group affected are largely men within the fertile age range (17). As a result of these physical morbidities, this young cohort of cancer survivors also exhibits demonstrably higher rates of depressive symptoms (34%) and depression and anxiety disorders (19% *vs*. 13.5%) compared with the general population (18–21).

The results derived from the quantitative aspects of this study describe and confirm the prevalence of previously reported side effects. The use of both the ED-5D-5L and the EORTC QLQ-TC26 gives insight into the landscape of side effects following different treatment modalities. Our qualitative analysis develops our understanding by identifying potentially “unasked” questions from a patient perspective that could ultimately improve post-treatment supportive care.

Our study included patients who received orchiectomy and a single dose adjuvant carboplatin. This treatment modality is an option for high-risk stage 1 seminomas (defined as being > 4 cm in maximum dimension and rete testis invasion (RTI)) to reduce this risk. Analysis of this group compared with other treatment groups suggested that patients had less anxiety related to possible cancer recurrence, with a very similar side-effect profile to those in the orchidectomy alone group. For some patients the provision of information that adjuvant treatment for stage 1 seminoma may offer a better quality of life, without impacting on their side-effect profile, may inform their decision as to which treatment to opt for.

Although quantitative analysis of the survey’s closed questions indicated that that a substantial proportion of individuals report ongoing health needs (22), these data do not shed light on the experiences of treatment aftercare, or on what might improve health outcomes or patient experiences. Nevertheless, the fact that more than half (41 out of 73) of participants took the opportunity to comment *via* the free-text questions provides a significant resource in itself. Although this cannot be viewed as “representative” of all patients, these data provide rich insights into the views of patients regarding the PROMs that are currently in use in TCa follow-up and the questions (some of them unasked) that have patient-weighted importance. These data are not available in other surveys or interview studies of TCa survivors published to date (23).

The methodology for this study, adopted alongside formal PROMs measures, demonstrates that individuals actively engage with the opportunity to provide comments related to their experiences, therefore providing data relating to which health outcomes should be reported in formal measures and illuminating the analysis of results from currently available questionnaires (24).

Despite the informative nature of this mixed-methods study, it has limitations. A single-center and single-country/language design introduces a selection bias that might limit the generalizability of results. In addition, as a single-site study, and despite the addition of a free-text section, there may well be many more side effects and therefore supportive care requirements that are yet to be disclosed by patients facing a lifetime of living with and beyond the consequences of their cancer treatment.

## Conclusions

This study reports the medium- to long-term HRQoL and side effects of TCa treatments, using both EQ-5D-5L and EORTC TC26 questionnaires, and identifies possibly “unasked” questions from a patient perspective in relation to supportive care needs following TCa treatment. These results may allow for improved patient counseling regarding side effects and QoL experienced following different treatment modalities. This can help to better inform patients, optimize the consenting process pre-treatment, and highlight patients’ potential post-treatment needs. Future research, particularly into the aforementioned “unasked” questions, may improve this further.

## Data availability statement

The raw data supporting the conclusions of this article will be made available by the authors, without undue reservation.

## Author contributions

Conception and design of the study: DN, AP, JK, and WC. Acquisition of data: AP. Analysis and interpretation of data: DN, WC, and JK. Drafting the article: WC and JK. Revising it critically for important intellectual content: all authors. Final approval of the version to be submitted: all authors. Agreement to be accountable for all aspects of the work in ensuring that questions related to the accuracy or integrity of any part of the work are appropriately investigated and resolved: DN, WC, and JK. All authors contributed to the article and approved the submitted version.
