# Supplementary material for: Understanding the health-related quality of life and treatment-related side-effects in patients who have been in remission from testicular cancer for 12–24 months
Source: Front Urol. 2023 Jul 31;3:1174626. doi: 10.3389/fruro.2023.1174626 (PMC12327304; doi:10.3389/fruro.2023.1174626)
Supplement: Supplementary file 1 [file Table_1.pdf]

*Supplementary Material*

# Article Title

## UNDERSTANDING THE HEALTH-RELATED QUALITY OF LIFE AND TREATMENT-RELATED SIDE-EFFECTS IN PATIENTS WHO HAVE BEEN IN REMISSION FROM TESTICULAR CANCER FOR 12-24 MONTHS

**Authors List: Walter Cazzaniga[1*], Janette Kinsella[1], Adam Kieran Pearce[2], Masood Moghul[1], Louis Fox[3], Mieke Van Hemelrijck[3],A. Reid[1,4], R. Huddart[1,4]David Nicol[1,4]**

**Affiliations:**

1. **Department of Uro-Oncology, The Royal Marsden NHS Foundation Trust, London, United Kingdom**
2. **Urology Dept, Royal Brisbane and Women's Hospital, Butterfield St, Herston, Brisbane, Qld 4006, Australia.**
3. **Centre for Cancer, Society, and Public Health, King's College London**
4. **Institute of Cancer Research, London, United Kingdom**

*** Correspondence:**
Walter Cazzaniga, MD. FEBU
Department of Urology,
The Royal Marsden NHS Foundation Trust,
203 Fulham Rd., London SW3 6JJ
Email: Walter.Cazzaniga@rmh.nhs.uk

**Questionnaires**

*EQ-5D-5L (rated 0=not, 1=yes)*

Q8. MOBILITY. Please select the answer which best describes your health TODAY

Q8.1. I have no problems in walking about

Q8.2. I have slight problems in walking about

Q8.3. I have moderate problems in walking about

Q8.4. I have severe problems in walking about

Q8.5. I am unable to walk about

Q9. SELF-CARE. Please select the answer which best describes your health TODAY.

Q9.1. I have no problems washing or dressing myself

Q9.2. I have slight problems washing or dressing myself

Q9.3. I have moderate problems washing or dressing myself

Q9.4. I have severe problems washing or dressing myself

Q9.5. I am unable to wash or dress myself

Q10. USUAL ACTIVITIES (eg work, study, housework, family or leisure activities). Please select the answer which best describes your health TODAY

Q10.1. I have no problems doing my usual activities

Q10.2. I have slight problems doing my usual activities

Q10.3. I have moderate problems doing my usual activities

Q10.4. I have severe problems doing my usual activities

Q10.5. I am unable to do my usual activities

Q11. PAIN / DISCOMFORT. Please select the answer which best describes your health TODAY

Q11.1. I have no pain or discomfort

Q11.2. I have slight pain or discomfort

Q11.3. I have moderate pain or discomfort

Q11.4. I have severe pain or discomfort

Q11.5. I have extreme pain or discomfort

Q12. ANXIETY / DEPRESSION. Please select the answer which best describes your health TODAY

Q12.1. I am not anxious or depressed

Q12.2. I am slightly anxious or depressed

Q12.3. I am moderately anxious or depressed

Q12.4. I am severely anxious or depressed

Q12.5. I am extremely anxious or depressed

Q13. We would like to know how good or bad your health is TODAY. This scale is numbered from 0 to 100.100 means the best health you can imagine.0 means the worst health you can imagine.Please move the slider to indicate how your health is TODAY.

VAS (visual analog scale - 0-100)

*EORTC-TC26 (rated 0=not at all, 1=a little, 2= quite a bit, 3= very much)*

Q14. Patients may experience the following symptoms or problems. Please indicate to what extent they affect you now.

Q14.1. Unwanted weight gain

Q14.2. Loss of muscle or reduced strength

Q14.3. Low libido or lack of an interest in sex

Q14.4. Difficulty getting or maintaining an erection

Q14.5. Concerns about ability to have children

Q15. Patients who have had testicular cancer treated may experience the following symptoms or problems. Please indicate to what extent you have experienced them during the past week (select the best answer for each question)

Q15.1. Tingling or numbness in your hands or feet

Q15.2. Pale or cold fingers or toes

Q15.3. Ringing in your ears (known as tinnitus)

Q15.4. Problems with hearing

Q15.5. Shortness of breath

Q15.6. Problems with sense of taste or smell

Q16. Patients who have had testicular cancer treated may experience the following symptoms or problems. Please indicate to what extent you have experienced them during the past week (select the best answer for each question)

Q16.1. Pain in stomach area

Q16.2. Problems with ejaculation (little or no semen coming out with orgasm)

Q16.3. Muscle twitching in the arms or legs

Q16.4. Loss of muscle in the legs

Q16.5. Loose bowel motions or diarrhoea

Q16.6. Concerns with body image

Q16.7. Skin problems (eg dry or itchy)

Q17. Patients who have had testicular cancer treated may experience the following symptoms or problems. Please indicate to what extent you have experienced them during the past week (select the best answer for each question

Q17.1. Feeling uncertain about the future

Q17.2. Feeling anxious about a possible recurrence of testicular cancer

Q17.3. Problems with job or education due to the disease or treatment

Q17.4. Problems finding someone to talk to about the disease

Q17.5. Financial problems due to the disease or treatment

Q17.6. Feeling less masculine as a result of the disease or treatment

**TABLES**

**Table1**

| | Orchidectomy | Orchidectomy + Carboplatin | Multi-agent Chemotherapy | PC-RPLND |
|---|---|---|---|---|
| n°(%) | 19 (26%) | 13 (18%) | 19 (26%) | 22 (30%) |
| Q8 | | | | |
| 1 | 19 (100.0) | 13 (100.0) | 18 (94.7) | 18 (81.8) |
| 2 | 0 (0.0) | 0 (0.0) | 0 (0.0) | 2 (9.1) |
| 3 | 0 (0.0) | 0 (0.0) | 1 (5.3) | 2 (9.1) |
| 4 | 0 (0.0) | 0 (0.0) | 0 (0.0) | 0 (0.0) |
| 5 | 0 (0.0) | 0 (0.0) | 0 (0.0) | 0 (0.0) |
| Q9 | | | | |
| 1 | 19 (100.0) | 13 (100.0) | 19 (100.0) | 20 (90.9) |
| 2 | 0 (0.0) | 0 (0.0) | 0 (0.0) | 1 (4.5) |
| 3 | 0 (0.0) | 0 (0.0) | 0 (0.0) | 1 (4.5) |
| 4 | 0 (0.0) | 0 (0.0) | 0 (0.0) | 0 (0.0) |
| 5 | 0 (0.0) | 0 (0.0) | 0 (0.0) | 0 (0.0) |
| Q10 | | | | |
| 1 | 17 (89.5) | 13 (100.0) | 12 (63.2) | 15 (68.2) |

| | | | | |
|---|---|---|---|---|
| 2 | 1 (5.3) | 0 (0.0) | 6 (31.6) | 5 (22.7) |
| 3 | 1 (5.3) | 0 (0.0) | 1 (5.3) | 2 (9.1) |
| 4 | 0 (0.0) | 0 (0.0) | 0 (0.0) | 0 (0.0) |
| 5 | 0 (0.0) | 0 (0.0) | 0 (0.0) | 0 (0.0) |
| Q11 | | | | |
| 1 | 15 (78.9) | 9 (69.2) | 11 (57.9) | 14 (63.6) |
| 2 | 3 (15.8) | 3 (23.1) | 4 (21.1) | 6 (27.3) |
| 3 | 1 (5.3) | 0 (0.0) | 2 (10.5) | 2 (9.1) |
| 4 | 0 (0.0) | 0 (0.0) | 2 (10.5) | 0 (0.0) |
| 5 | 0 (0.0) | 1 (7.7) | 0 (0.0) | 0 (0.0) |
| Q12 | | | | |
| 1 | 8 (42.1) | 9 (69.2) | 10 (55.6) | 9 (40.9) |
| 2 | 7 (36.8) | 3 (23.1) | 4 (22.2) | 7 (31.8) |
| 3 | 4 (21.1) | 0 (0.0) | 3 (16.7) | 4 (18.2) |
| 4 | 0 (0.0) | 1 (7.7) | 1 (5.6) | 1 (4.5) |
| 5 | 0 (0.0) | 0 (0.0) | 0 (0.0) | 1 (4.5) |

**Table1:** Results obtained from the ED-5D-5L Questionnaire; Levels: 1=No problem, 2=slight problem, 3=moderate problem, 4=severe problem, 5=extreme problem; Full text questions are reported as a supplementary material

**Table2**

| | Orchidectomy | Orchidectomy + Carboplatin | Multi-agent Chemotherapy | PC-RPLND |
|---|---|---|---|---|
| VAS<br>Median (IQR) | 80 (75-90.5) | 90 (75-95) | 75 (70-87) | 75 (75-90) |

**Table2:** VAS stratified according to treatment group; IQR: Interquartile range

**Table3**

| | Orchidectomy | Orchidectomy + Carboplatin | Multi-agent Chemotherapy | PC-RPLND |
|---|---|---|---|---|
| n°(%) | 19 (26%) | 13 (18%) | 19 (26%) | 22 (30%) |
| Q14.1 | | | | |
| 0 | 10 (52.6) | 8 (61.5) | 10 (55.6) | 11 (50.0) |
| 1 | 5 (26.3) | 4 (30.8) | 4 (22.2) | 6 (27.3) |
| 2 | 3 (15.8) | 1 (7.7) | 2 (11.1) | 4 (18.2) |
| 3 | 1 (5.3) | 0 (0.0) | 2 (11.1) | 1 (4.5) |
| Q14.2 | | | | |
| 0 | 10 (58.8) | 9 (69.2) | 6 (31.6) | 5 (22.7) |
| 1 | 5 (29.4) | 3 (23.1) | 4 (21.1) | 13 (59.1) |
| 2 | 2 (11.8) | 1 (7.7) | 7 (36.8) | 4 (18.2) |
| 3 | 0 (0.0) | 0 (0.0) | 2 (10.5) | 0 (0.0) |
| Q14.3 | | | | |
| 0 | 9 (47.4) | 8 (61.5) | 13 (68.4) | 13 (59.1) |
| 1 | 7 (36.8) | 5 (38.5) | 3 (15.8) | 7 (31.8) |
| 2 | 2 (10.5) | 0 (0.0) | 1 (5.3) | 1 (4.5) |
| 3 | 1 (5.3) | 0 (0.0) | 2 (10.5) | 1 (4.5) |
| Q14.4 | | | | |
| 0 | 14 (73.7) | 12 (92.3) | 12 (63.2) | 16 (72.7) |
| 1 | 3 (15.8) | 1 (7.7) | 5 (26.3) | 4 (18.2) |
| 2 | 1 (5.3) | 0 (0.0) | 1 (5.3) | 0 (0.0) |
| 3 | 1 (5.3) | 0 (0.0) | 1 (5.3) | 2 (9.1) |
| Q14.5 | | | | |

| | | | | |
|---|---|---|---|---|
| 0 | 11 (57.9) | 12 (92.3) | 12 (63.2) | 12 (54.5) |
| 1 | 5 (26.3) | 0 (0.0) | 4 (21.1) | 3 (13.6) |
| 2 | 2 (10.5) | 1 (7.7) | 3 (15.8) | 2 (9.1) |
| 3 | 1 (5.3) | 0 (0.0) | 0 (0.0) | 5 (22.7) |
| Q15.1 | | | | |
| 0 | 16 (84.2) | 10 (76.9) | 6 (31.6) | 10 (45.5) |
| 1 | 2 (10.5) | 2 (15.4) | 8 (42.1) | 5 (22.7) |
| 2 | 1 (5.3) | 1 (7.7) | 2 (10.5) | 3 (13.6) |
| 3 | 0 (0.0) | 0 (0.0) | 3 (15.8) | 4 (18.2) |
| Q15.2 | | | | |
| 0 | 15 (78.9) | 12 (92.3) | 9 (47.4) | 15 (68.2) |
| 1 | 4 (21.1) | 0 (0.0) | 5 (26.3) | 1 (4.5) |
| 2 | 0 (0.0) | 1 (7.7) | 3 (15.8) | 3 (13.6) |
| 3 | 0 (0.0) | 0 (0.0) | 2 (10.5) | 3 (13.6) |
| Q15.3 | | | | |
| 0 | 15 (78.9) | 11 (84.6) | 11 (61.1) | 11 (50.0) |
| 1 | 2 (10.5) | 2 (15.4) | 2 (11.1) | 5 (22.7) |
| 2 | 1 (5.3) | 0 (0.0) | 1 (5.6) | 5 (22.7) |
| 3 | 1 (5.3) | 0 (0.0) | 4 (22.2) | 1 (4.5) |
| Q15.4 | | | | |
| 0 | 15 (78.9) | 11 (84.6) | 9 (47.4) | 13 (59.1) |
| 1 | 4 (21.1) | 2 (15.4) | 8 (42.1) | 7 (31.8) |
| 2 | 0 (0.0) | 0 (0.0) | 1 (5.3) | 1 (4.5) |
| 3 | 0 (0.0) | 0 (0.0) | 1 (5.3) | 1 (4.5) |
| Q15.5 | | | | |
| 0 | 15 (78.9) | 11 (84.6) | 10 (52.6) | 10 (45.5) |

| | | | | |
|---|---|---|---|---|
| 1 | 4 (21.1) | 2 (15.4) | 6 (31.6) | 9 (40.9) |
| 2 | 0 (0.0) | 0 (0.0) | 2 (10.5) | 2 (9.1) |
| 3 | 0 (0.0) | 0 (0.0) | 1 (5.3) | 1 (4.5) |
| Q15.6 | | | | |
| 0 | 16 (84.2) | 11 (84.6) | 13 (68.4) | 19 (86.4) |
| 1 | 1 (5.3) | 2 (15.4) | 5 (26.3) | 2 (9.1) |
| 2 | 1 (5.3) | 0 (0.0) | 1 (5.3) | 1 (4.5) |
| 3 | 1 (5.3) | 0 (0.0) | 0 (0.0) | 0 (0.0) |
| Q16.1 | | | | |
| 0 | 17 (89.5) | 12 (92.3) | 12 (63.2) | 14 (63.6) |
| 1 | 1 (5.3) | 0 (0.0) | 4 (21.1) | 7 (31.8) |
| 2 | 1 (5.3) | 1 (7.7) | 2 (10.5) | 0 (0.0) |
| 3 | 0 (0.0) | 0 (0.0) | 1 (5.3) | 1 (4.5) |
| Q16.2 | | | | |
| 0 | 16 (84.2) | 12 (92.3) | 15 (78.9) | 8 (36.4) |
| 1 | 3 (15.8) | 1 (7.7) | 4 (21.1) | 2 (9.1) |
| 2 | 0 (0.0) | 0 (0.0) | 0 (0.0) | 0 (0.0) |
| 3 | 0 (0.0) | 0 (0.0) | 0 (0.0) | 12 (54.5) |
| Q16.3 | | | | |
| 0 | 16 (84.2) | 12 (92.3) | 13 (68.4) | 17 (77.3) |
| 1 | 3 (15.8) | 0 (0.0) | 2 (10.5) | 4 (18.2) |
| 2 | 0 (0.0) | 0 (0.0) | 4 (21.1) | 1 (4.5) |
| 3 | 0 (0.0) | 1 (7.7) | 0 (0.0) | 0 (0.0) |
| Q16.4 | | | | |
| 0 | 16 (84.2) | 11 (84.6) | 12 (63.2) | 17 (77.3) |
| 1 | 3 (15.8) | 2 (15.4) | 6 (31.6) | 4 (18.2) |
| 2 | 0 (0.0) | 0 (0.0) | 0 (0.0) | 1 (4.5) |

| | | | | |
|---|---|---|---|---|
| 3 | 0 (0.0) | 0 (0.0) | 1 (5.3) | 0 (0.0) |
| Q16.5 | | | | |
| 0 | 16 (84.2) | 11 (84.6) | 15 (78.9) | 17 (77.3) |
| 1 | 3 (15.8) | 1 (7.7) | 4 (21.1) | 4 (18.2) |
| 2 | 0 (0.0) | 1 (7.7) | 0 (0.0) | 1 (4.5) |
| 3 | 0 (0.0) | 0 (0.0) | 0 (0.0) | 0 (0.0) |
| Q16.6 | | | | |
| 0 | 10 (52.6) | 8 (61.5) | 9 (47.4) | 10 (45.5) |
| 1 | 5 (26.3) | 4 (30.8) | 5 (26.3) | 8 (36.4) |
| 2 | 3 (15.8) | 0 (0.0) | 3 (15.8) | 2 (9.1) |
| 3 | 1 (5.3) | 1 (7.7) | 2 (10.5) | 2 (9.1) |
| Q16.7 | | | | |
| 0 | 15 (78.9) | 10 (76.9) | 13 (68.4) | 14 (63.6) |
| 1 | 1 (5.3) | 2 (15.4) | 4 (21.1) | 7 (31.8) |
| 2 | 2 (10.5) | 0 (0.0) | 1 (5.3) | 1 (4.5) |
| 3 | 1 (5.3) | 1 (7.7) | 1 (5.3) | 0 (0.0) |
| Q17.1 | | | | |
| 0 | 6 (42.9) | 8 (61.5) | 5 (38.5) | 5 (38.5) |
| 1 | 8 (57.1) | 4 (30.8) | 7 (53.8) | 7 (53.8) |
| 2 | 0 (0.0) | 0 (0.0) | 0 (0.0) | 0 (0.0) |
| 3 | 0 (0.0) | 1 (7.7) | 1 (7.7) | 1 (7.7) |
| Q17.2 | | | | |
| 0 | 5 (33.3) | 3 (25.0) | 3 (27.3) | 3 (20.0) |
| 1 | 10 (66.7) | 7 (58.3) | 7 (63.6) | 11 (73.3) |
| 2 | 0 (0.0) | 0 (0.0) | 0 (0.0) | 0 (0.0) |
| 3 | 0 (0.0) | 2 (16.7) | 1 (9.1) | 1 (6.7) |

| | | | | |
|---|---|---|---|---|
| Q17.3 | | | | |
| 0 | 12 (75.0) | 12 (92.3) | 9 (69.2) | 11 (64.7) |
| 1 | 4 (25.0) | 1 (7.7) | 4 (30.8) | 4 (23.5) |
| 2 | 0 (0.0) | 0 (0.0) | 0 (0.0) | 0 (0.0) |
| 3 | 0 (0.0) | 0 (0.0) | 0 (0.0) | 2 (11.8) |
| Q17.4 | | | | |
| 0 | 14 (82.4) | 11 (84.6) | 10 (62.5) | 11 (61.1) |
| 1 | 3 (17.6) | 0 (0.0) | 5 (31.2) | 6 (33.3) |
| 2 | 0 (0.0) | 0 (0.0) | 0 (0.0) | 0 (0.0) |
| 3 | 0 (0.0) | 2 (15.4) | 1 (6.2) | 1 (5.6) |
| Q17.5 | | | | |
| 0 | 14 (82.4) | 12 (92.3) | 12 (75.0) | 12 (66.7) |
| 1 | 2 (11.8) | 1 (7.7) | 2 (12.5) | 4 (22.2) |
| 2 | 0 (0.0) | 0 (0.0) | 0 (0.0) | 0 (0.0) |
| 3 | 1 (5.9) | 0 (0.0) | 2 (12.5) | 2 (11.1) |

**Table3:** Results obtained from the EORTC QLQ-TC26 Questionnaire; Levels: 0=not at all, 1=a little, 2= quite a bit, 3= very much; Full text questions are reported as a supplementary material

**Table4**: Symptoms or problems not mentioned on the form

| Symptom | Freetext |
|---|---|
| **Body image** | Not a big deal, but I am a bit self-conscious about the prosthesis |
| | 1.Hair thinning which has not improved - potentially due to genetic hair loss in family.<br>2.Permanent pigmentation on body after chemotherapy<br>3.Toe and finger nail damaged.<br>4. I suffered from incomplete regrowth of hair |
| **Pain** | 1.Occasional aching in groin area, which I appreciate is down to the operation. 2.Doesn't stop me doing anything, but is sometimes quite uncomfortable. |
| | 1. Pain daily at night.<br>2. When i cum i get a pain within the testicular area that has been removed<br>3. Slight pain/discomfort when ejaculating.<br>3. A feeling of tightness under the scar tissue from surgery.<br>4. The scar line of the surgery, ever so light numbness over the incision and surrounding area, but not noticeable unless you touch it directly.<br>5. Slight pain/discomfort in right leg (right sided testicle removed.<br>6. Used to have a pain in the same area when ejaculating but this has recently been less painful and more infrequent<br>7. Discomfort in wound site from orchiectomy |
| | 1. I still sometimes get a tingling feeling in the testis area<br>2. I often get an electric shock sensation in my chest arms and legs |
| | 1. Pain in diaphragm stemming from placating it during surgery<br>2. Discomfort in wound site from orchiectomy<br>3.Raynaud's affecting fingers<br>4.Raynaud's disease much worse after chemotherapy. |
| **Shortness of breath** | 1.I often feel breathless while resting .<br>2.A little chesty first thing in the morning but quickly goes away |
| | 1. Weak lungs.<br>2.So hard to breath |
| **Anxiety** | I had my left bollock removed and around the groin of my right one, when examined by myself and later by a physician feels knotty just a little. I think this maybe the lymph glands. These have been checked out but still causes some anxiety. |
| | I suffer from claustrophobia now |

| | |
|---|---|
| | |
| | 1. Whilst I'm not a worrier and have a healthy attitude to getting on with it in life, I do wonder how if at all, how the Chemotherapy has affected me |
| **Cognitive impairment** | 1.It was 1 year after chemo before I felt 'normal' and back to my old self.<br>2.Suffered from mild cognitive impairment for almost a year. |
| | Memory recall, e.g. people's names. Not finding the right word as easily as before. 2.Concentration and speed of thought. |

**Key:**

▲ Orchidectomy only

▲ Orchidectomy + Carboplatin

▲ RPLND

▲ Chemotherapy

**Table5:** Single aspect of TCa that has the greatest impact on your life

| Symptom | Freetext |
|---|---|
| Body image | 1.I seem to have developed fatty chest tissue which I am very self-conscious about.<br>2.I feel less of a man and I had already had the snip, so having more children wasn't an issue. But never the less I feel less confident and a shadow of my former self. |
| | 1.I am slightly shy of the massive scar on my tummy in public, not that it's a big deal. Very small price to pay.<br>2.I now have an incisional hernia<br>3. Removal of one of my testis and being unable to produce semen affected my masculinity. |
| Emotional impact of cancer | 1. The single aspect would be the lingering thought that it may return.<br>2. Increased stress from something else to worry about<br>3. Mental impact - not knowing the true cause and the likelihood of it returning.<br>4. I'd like to think I'm learning to deal with it better as each month passes and my results are remain clear, I'm now being monitored every 3 months rather than every month |
| | 1. When I go to the cancer centre for check-ups I see people suffering infinitely worse than I did. So I almost feel like I don't have the right to talk about it or to acknowledge it happened to me.<br>2.The fact you had it once always makes you anxious any things especially during check up time. So its more a mental impact than physical<br>3. I have a 2 year old and another baby due in January 2019 (conceived 18 months after treatment - yey!) and I worry about long term life expectancy. |
| | 1.A low level anxiety about a possible future recurrence.<br>2.Low mood, feeling rundown with cold like symptoms most of the time |
| | 1.Uncertainty for future.<br>2.Would be useful to have statistics of how likely it is to reoccur.<br>3. I can't be clean shaven or have short hair, if I do I can't look at myself in a mirror as it reminds me of being ill.<br>4.The surgery was on sensitive parts, and the feeling doesn't go away. I guess it's all psychological impact<br>5.I'm constantly worried; I have financial problems due to being dismissed at work for being off sick, and now have depression and a fear this wont end |
| | 1. The fact that CT scans, radiotherapy and chemotherapy are all associated with increased risk of cancer in later life. So just a low-level of uncertainty in that respect.<br>2.I feel anxious about the possibility of having cancer again |
| Fatigue | Suffering from low energy levels from time to time possibly due to low/borderline testosterone level. |

| | |
|---|---|
| | 1.I found it hard to get back into exercise. Only now almost two years after diagnosis have I managed to start doing proper regular exercise.<br>2.Lack of energy resulting in inactivity and weight gain |
| | 1.I Constantly feel tired , And have problems sleeping , due to the feeling of laying on a ball when laying on my side<br>2.lack of energy and poor general fitness. |
| | The fatigue is overwhelming |

**Key:**

▲ Orchidectomy only

▲ Orchidectomy + Carboplatin

▲ RPLND

▲ Chemotherapy
